# Supplementary material for: Ferroelectricity in graphene nanoribbon devices enabled by collective water molecule dynamics
Source: Nat Commun. 2025 Dec 9;16:10982. doi: 10.1038/s41467-025-65922-6 (PMC12689630; doi:10.1038/s41467-025-65922-6)
Supplement: Supplementary file 1 — Supplementary Information [file 41467_2025_65922_MOESM1_ESM.pdf]

SUPPORTING INFORMATION

Ferroelectricity in Graphene Nanoribbon Devices Enabled by Collective Water Molecule Dynamics

Muhammad Awais Aslam[1,†], *Igor Stanković*[1,2,†,*], *Gennadiy Murastov*[1], *Amy Carl*[3,4], *Muhammad Zubair Khan*[1], *Zehao Song*[5], *Kenji Watanabe*[6], *Takashi Taniguchi*[7], *Alois Lugstein*[5], *Christian Teichert*[1], *Roman Gorbachev*[3], *Raul D. Rodriguez*[8,*], *and Aleksandar Matković*[1,*]

[1] Chair of Physics, Department Physics, Mechanics, and Electrical Engineering, Montanuniversität Leoben, Franz Josef Strasse 18, 8700 Leoben, Austria.
[2] Scientific Computing Laboratory, Center for the Study of Complex Systems, Institute of Physics Belgrade, University of Belgrade, 11080 Belgrade, Serbia.
[3] Department of Physics and Astronomy and National Graphene Institute, University of Manchester, M13 9PL Manchester, United Kingdom.
[4] Department of Materials and National Graphene Institute, University of Manchester, M13 9PL Manchester, United Kingdom.
[5] Institute of Solid State Electronics, TU Wien, Gußhausstraße 25-25a, 1040 Vienna, Austria.
[6] Research Center for Electronic and Optical Materials, National Institute for Materials Science, 1- 1 Namiki, Tsukuba 305-0044, Japan.
[7] Research Center for Materials Nanoarchitectonics, National Institute for Materials Science, 1-1 Namiki, Tsukuba, 305-0044, Japan.
[8] Tomsk Polytechnic University, Lenina ave. 30, 634034, Tomsk, Russia.

† equal contribution
* igor@ipb.ac.rs, aleksandar.matkovic@unileoben.ac.at, raul@tpu.ru

Table of Content:

- S1: Fabrication scheme
- S2: Nanoribbon widths estimated by AFM
- S3: Scheme of the potential charge-trap sources in the fabricated NR FETs and AFM images of the organic nanostructures versus the interfacial bubbles
- S4: Comparison between the electrical transfer curves
- S5: Statistics from the entire device dataset for the peak apparent electron mobility values
- S6: Examples of the mobility versus gate bias curves
- S7: Statistics from the entire device dataset for the hysteresis window (V_H)
- S8: Individual Arrhenius function-based fits for the $V_H(T)$ mono-layer curves
- S9: V_H dependence on the V_{SG} sweeping range
- S10: Details of dipole directionality
- S11: Device annealing sequence
- S12: Comparison between V_H in air and under vacuum
- S13: Mechanistic illustration of the self-stabilizing effect on the bi stable system
- S14: Influence of the water molecule density on the induced field and water cluster stability
- S15: Distribution of the water molecules along the ribbon edges
- S16: Estimate of the field enhancement due to the fringing capacitance effect
- S17: Field distribution from the MD simulation over the entire $2L$ NR width
- S18: Overview of the MD model
- S19: Dipole induced field decay as a function of the distance along the ribbon width direction
- S20: A comparison between symmetric and asymmetric gate bias sweeping

S1: Fabrication scheme

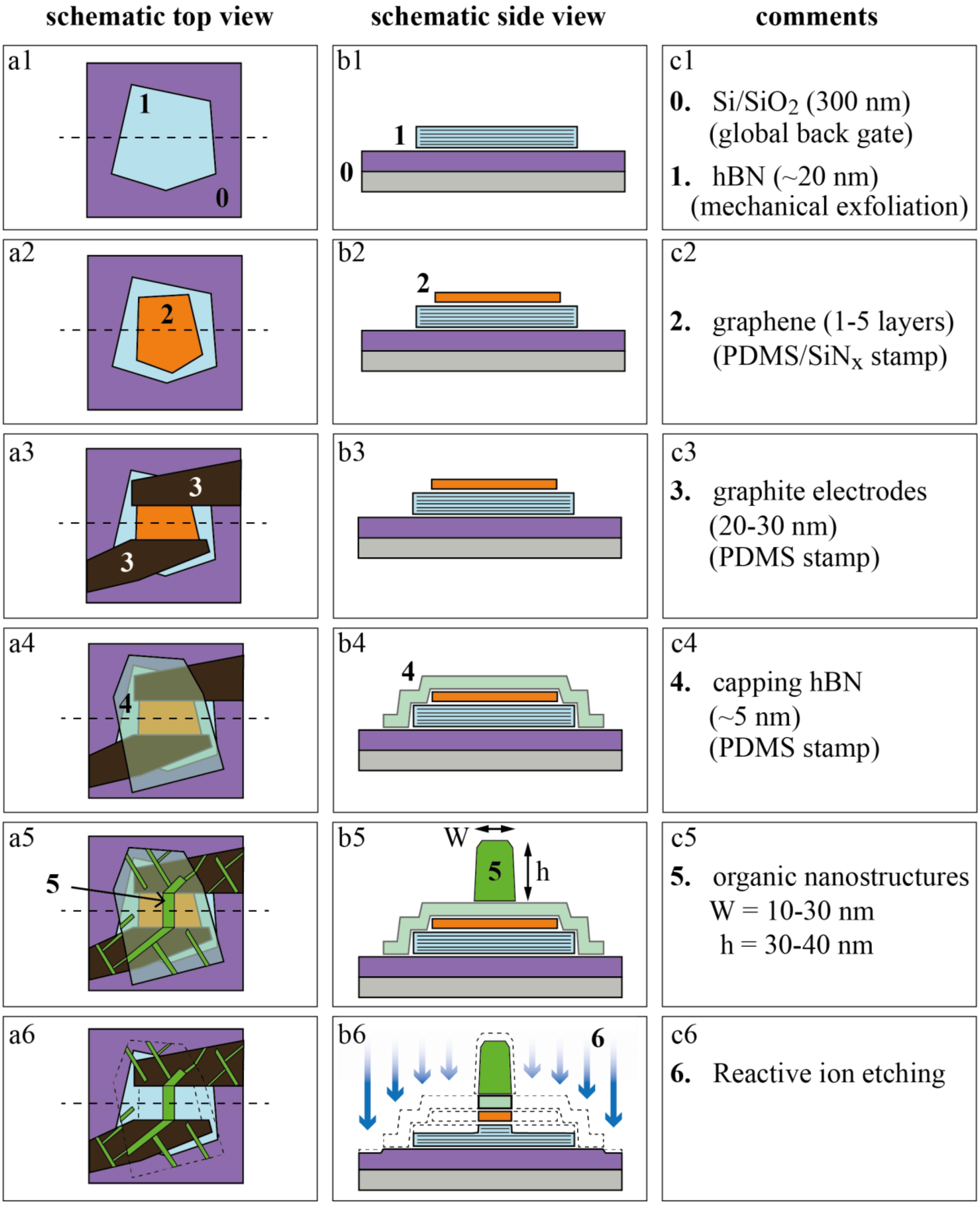

Figure S1: (a1-6) schematic top view of the fabrication steps. (b1-6) schematic side view of the fabrication steps, presenting corresponding cross-sections labelled in (a1-6) with a horizontal dashed line. Mind, the schematic presentations are not to scale. Numbers "0" to "6" indicate individual layers or fabrication steps. These are labelled in (c1-6) also providing the additional comments on their key parameters. For the sake of clarity, in the steps "5" and "6" only one nanostructure/nanoribbon is presented bridging the electrodes. This is not the case in the fabricated devices, as usually multiple ribbons with several ribbon junctions cross the channel.

S2: Nanoribbon widths estimated by AFM

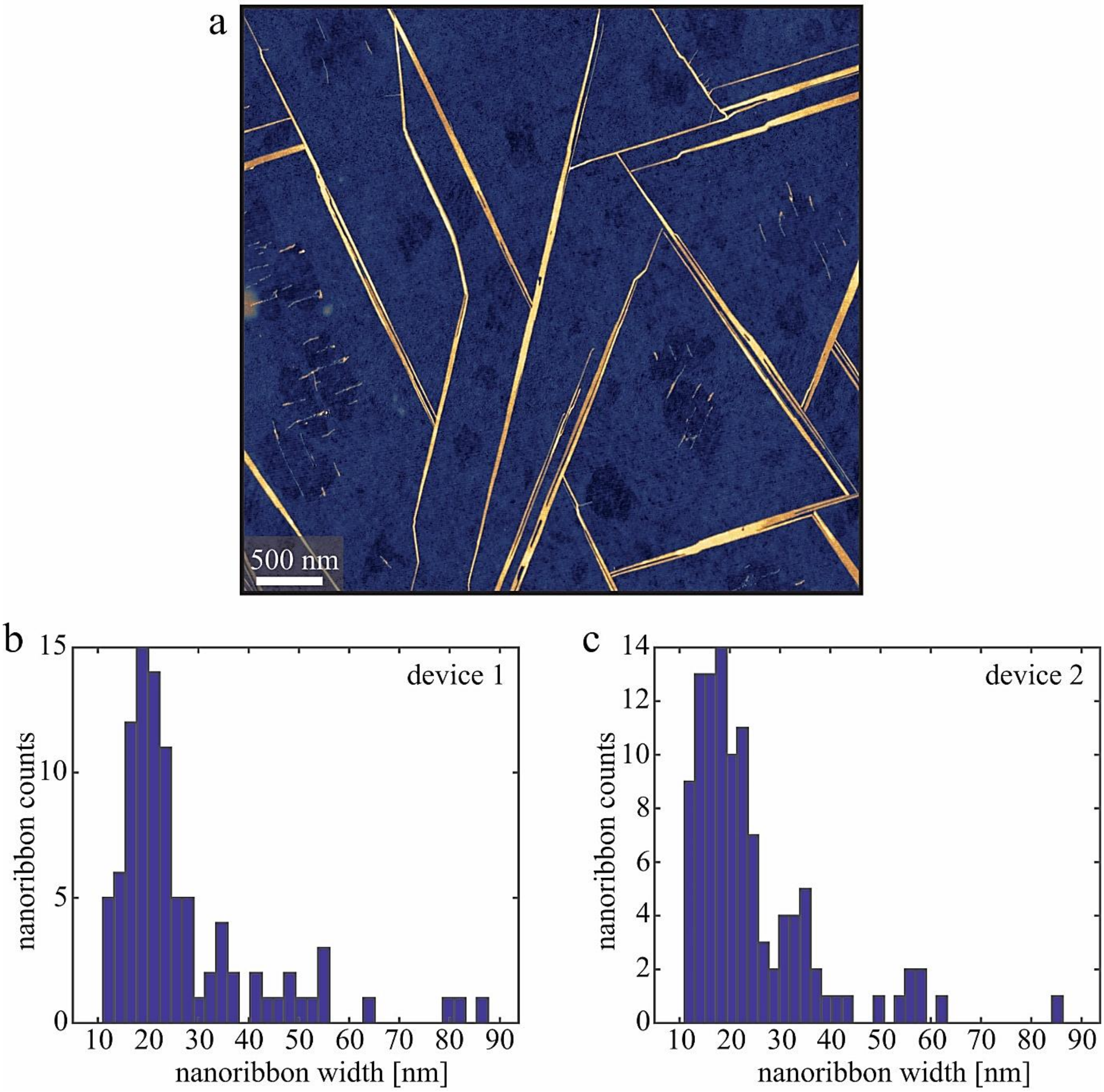

Figure S2: (a) high-resolution AFM topography of a graphene nanoribbon network (*z* scale 8 nm, recorded pixel size 2.6 nm). (b, c) nanoribbon width statistics for two independently fabricated nanoribbon devices, in each case considering 100 nanoribbons.

S3: Scheme of the potential charge trap sources in the fabricated NR FETs and AFM images of the organic nanostructures versus the interfacial bubbles

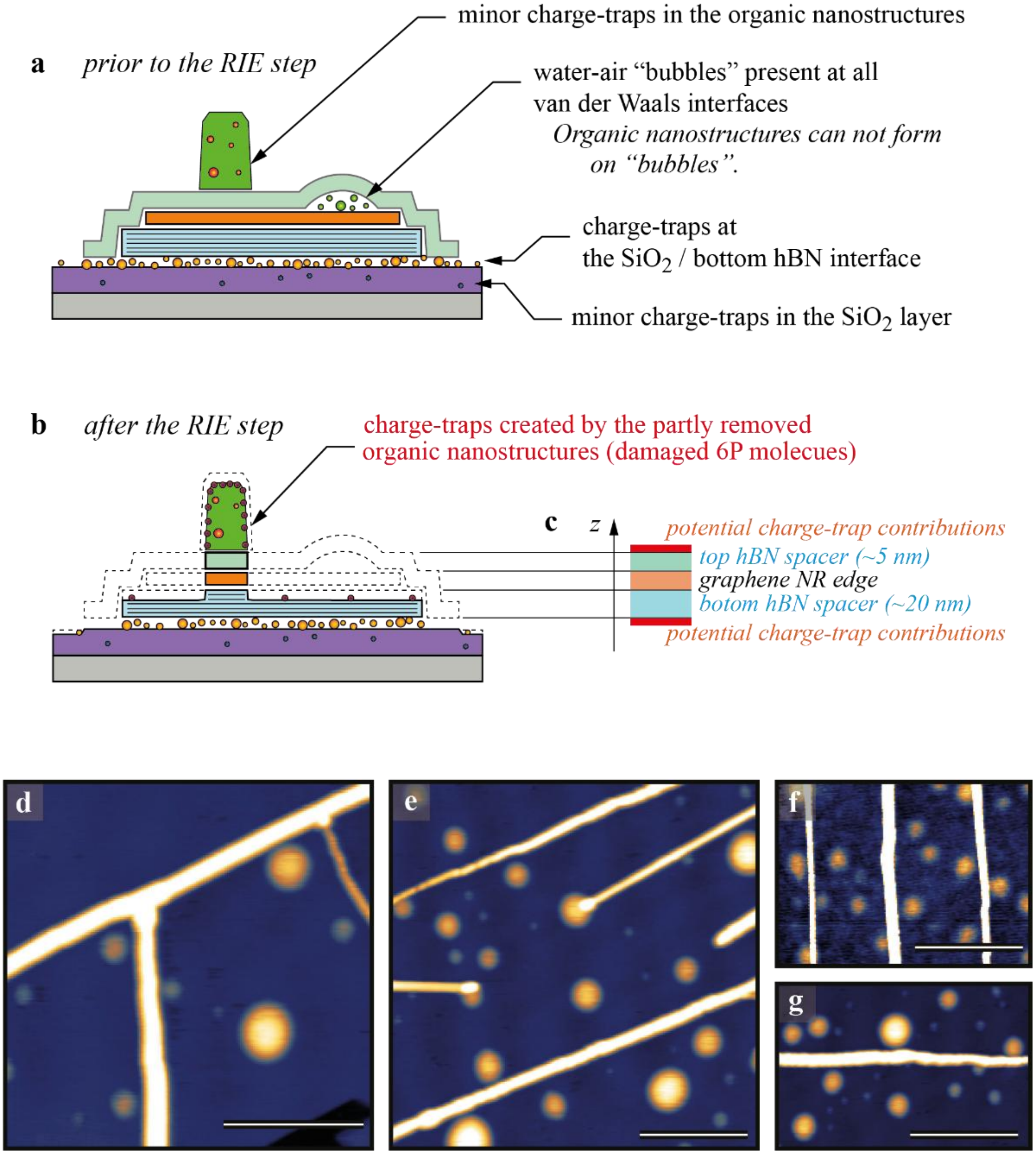

Figure S3: (a) schematic cross-section of the stack after formation of the organic nanostructures. Spheres indicate potential charge trap sources. (b) schematic cross-section after the reactive ion etching (RIE) step and the formation of the hBN/graphene/hBN nanoribbons. (c) Cross-sectional illustration of the layer stack (not to scale) (d-g) AFM topography images of the nanoribbon networks before the RIE step (scale bar 500 nm, *z* scale 30 nm). Observed bubbles are characteristic interfacial traps of water-air mixture within the layers of the van der Waals hetero stack (hBN/graphene/hBN). Bi axial strain of the top layer induced by the bubbles hinders the self-assembly of the organic nanostructures. Consequently, the organic nanostructures tend to form between the bubbles or tend to terminate on the bubbles.

S4. Comparison between the electrical transfer curves

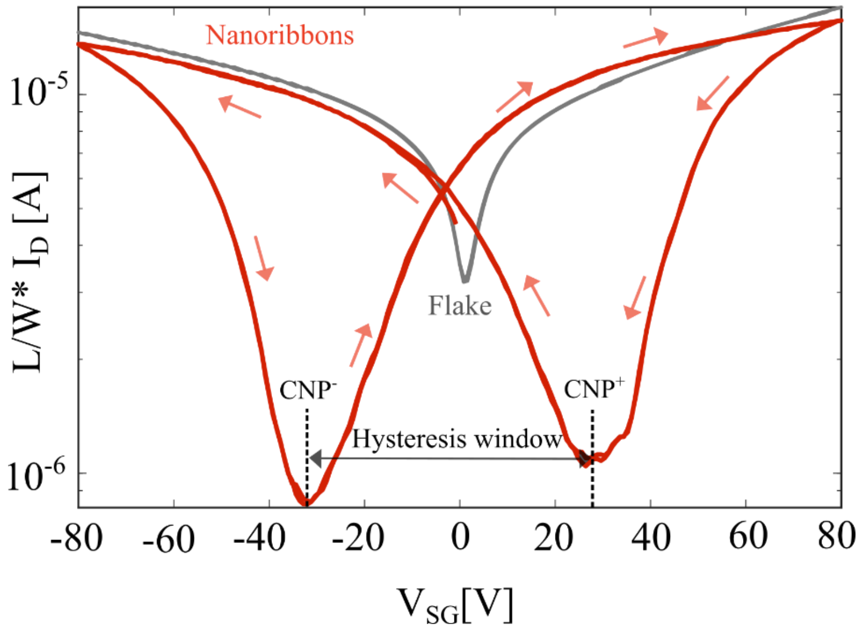

Figure S4: Semi-log scale electrical transfer curves of the same 1*L* graphene device before and after being patterned into the network of nanoribbons. The measurements were carried out at 300 K under $2{\cdot}10^{-2}$ mbar, and two subsequent sweeps are presented. I_D is normalized with respect to the length (*L*) over width (*W*) ratio of the flake and the nanoribbon network, respectively. The geometry of the nanoribbon channel was determined by the AFM. Unpolarized position of the CNP in the case of the nanoribbon network was estimated at -4 V.

S5: Statistics from the entire device dataset for the peak apparent electron mobility values

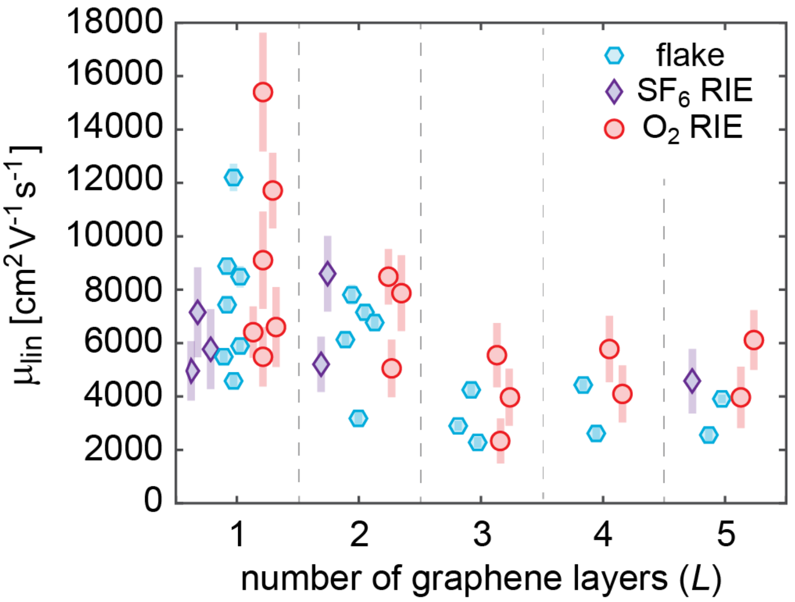

Figure S5: Device statistics for the linear apparent electron mobility as a function of the number of graphene layers $\mu_{lin}(L)$. The values are extracted as peak mobilities from the electrical transfer curves recorded at 300 K ($2 \cdot 10^{-2}$ mbar). The total of 22 devices that were used in the study is presented. Hexagons correspond to the flake FETs before the RIE step (step 5 in the fabrication scheme – Figure S1), diamonds represent Gr NR FETs etched using SF_6 RIE, and circles using O_2 RIE. Mobilities are extracted in all cases using a linear regime approximation and a parallel capacitance model. Fringing capacitance model for the NR FETs results in about one order of magnitude lower values. Length and width of each device were determined via a combination of the optical micrographs and AFM topography images. The uncertainty is estimated as a standard deviation considering five subsequent $I_D(V_{SG})$ sweeps and the deviations in the length and width of the ribbon networks for each device. The larger uncertainty for the Gr NR FETs originates from the uncertainty of the NR widths measured by the AFM.

S6: Examples of the mobility versus gate bias curves

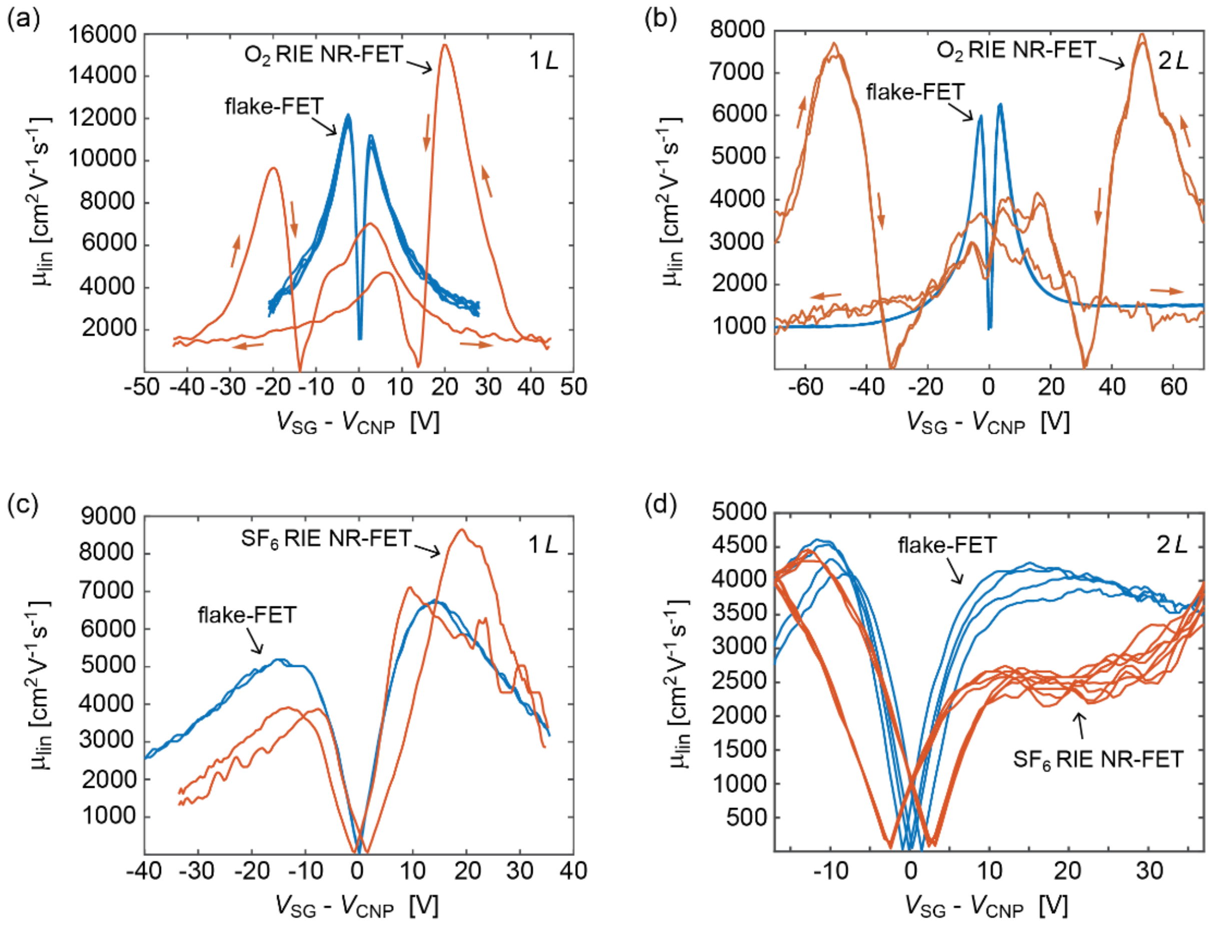

Figure S6: Linear apparent electron mobility as a function of gate voltage. Each sub-panel compares the $\mu_{\mathrm{lin}}(V_{\mathrm{SG}})$ curves of devices before and after the RIE step (labeled as flake- and NR-FETs, respectively). The *x*-axis is compensated for the V_{CNP} values for better comparison. The curves are obtained from the electrical transfer data recorded at 300 K ($2{\cdot}10^{-2}$ mbar) using the linear regime parallel capacitance mobility model. (a) and (b) present two devices with O_2 RIE etching of 1 and 2 graphene layers, respectively. (c) and (d) comparable devices to (a) and (b), only with SF_6 RIE step.

S7: Statistics from the entire device dataset for the hysteresis window (V_H)

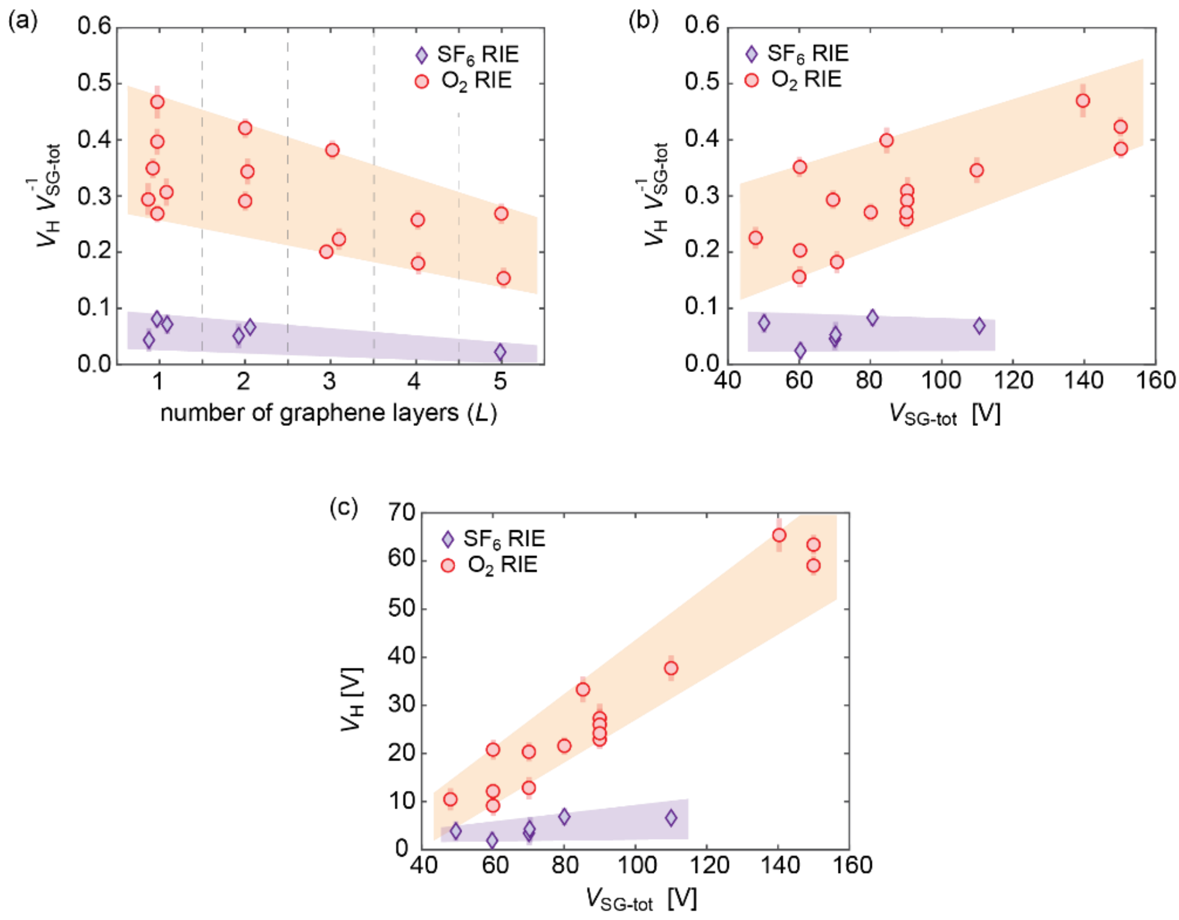

Figure S7: Statistics of the hysteresis window (V_H) considering the entire set of devices used in the study (16 O_2 RIE and 6 SF_6 RIE FETs). Red circles represent O_2 etched devices and purple diamonds represent SF_6 etched ones. Shaded areas serve as a guide to the eye to indicate the trends and the device-to-device scattering of the parameters. The data are extracted from the electrical transfer curves measured at 300 K and under $2 \cdot 10^{-2}$ mbar. Each data point with its error bar is extracted as a mean and standard deviation from five transfer curves. (a,b) V_H is scaled by the total V_{SG} sweeping range ($V_{SG\text{-}tot}$), as a function of the number of graphene layers (a) and as a function of $V_{SG\text{-}tot}$ (b). (c) presents $V_H(V_{SG\text{-}tot})$ without scaling. Likely, the main source for the device-to-device scatter is the variation in the width of the nanoribbons in the self-assembled networks.

S8: Individual Arrhenius function-based fits for the $V_H(T)$ mono-layer curves

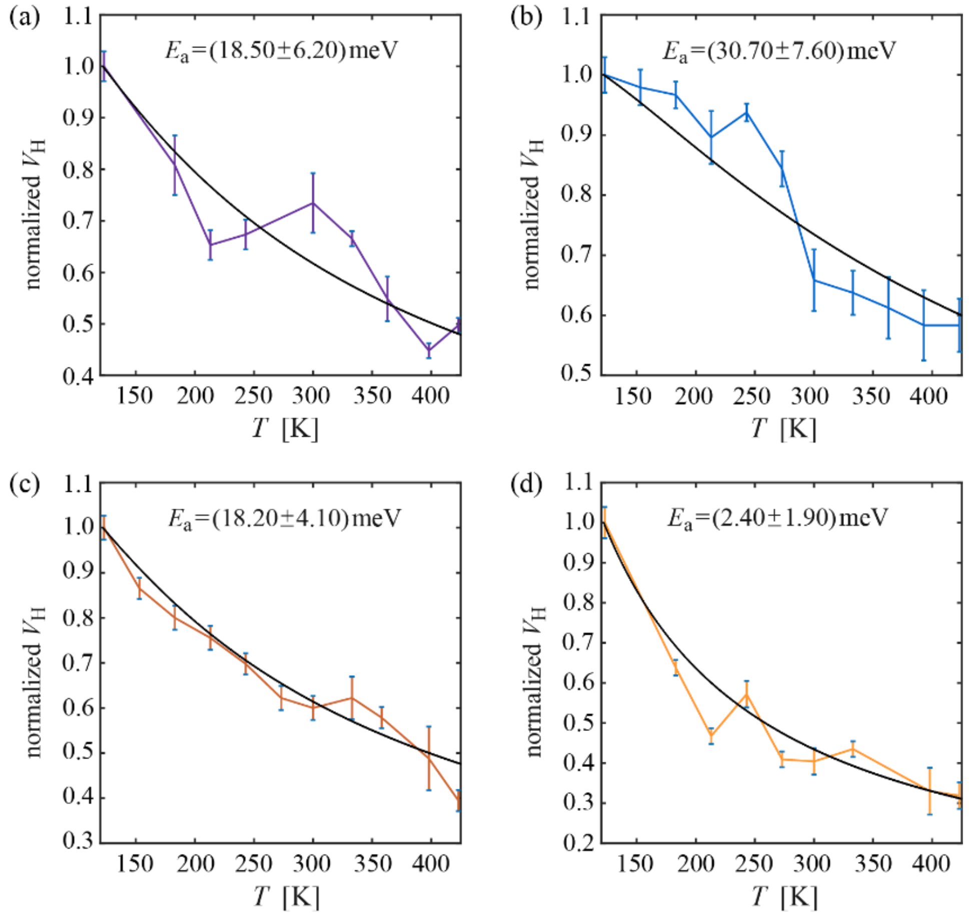

Figure S8. (a-d) separately fitted Arrhenius equation-based model of the energy barrier height for each of the mono-layer devices from Figure 1e of the main manuscript. The fitting is carried out by the root mean square minimization and the uncertainties imply a standard deviation. Error bars for the experimental points indicate a standard deviation of V_H extracted from five subsequent $I_D(V_{SG})$ sweeps.

S9: V_H dependence on the V_{SG} sweeping range

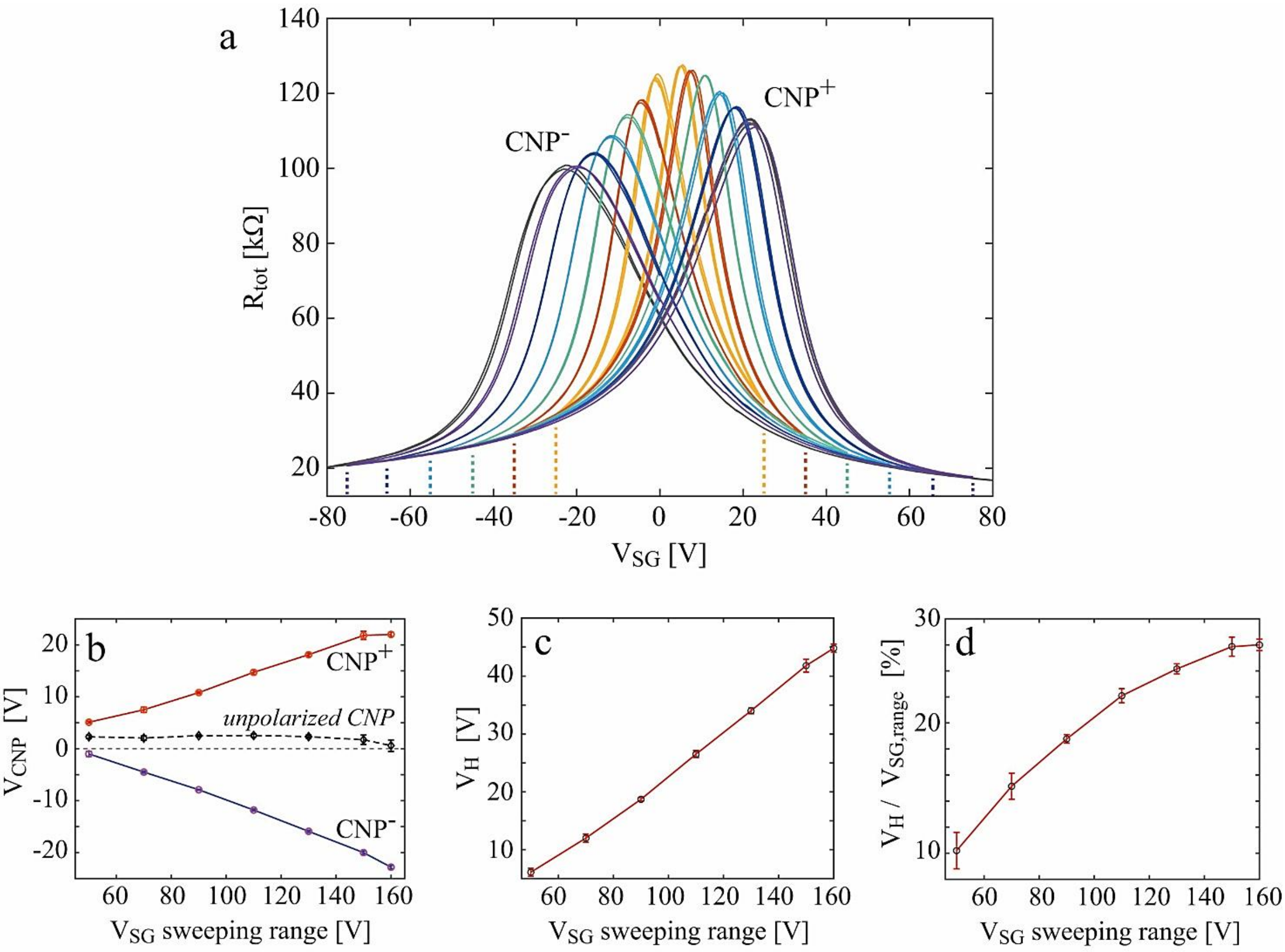

Figure S9: (a) total resistance transfer curves of a 4L Gr NR FET ($2\cdot10^{-2}$ mbar, 300 K) with subsequently increasing V_{SG} sweeping range. Between each measurements the device was left to relax to the unpolarized state. Dashed vertical lines indicate the sweeping ranges of the corresponding transfer curves. (b) extracted positions of the $CNP^{\pm}$ as a function of the total V_{SG} sweeping range. The unpolarized CNP was estimated from the initial curves (not plotted in a for clarity). The shift of the curves towards positive bias indicates minor unintentional *p*-type doping of the device. (c) a total hysteresis window $V_H = |V_{CNP+}| + |V_{CNP-}|$. d fraction of the total hysteresis V_H with respect to the total V_{SG} sweeping range.

S10. Details of dipole directionality

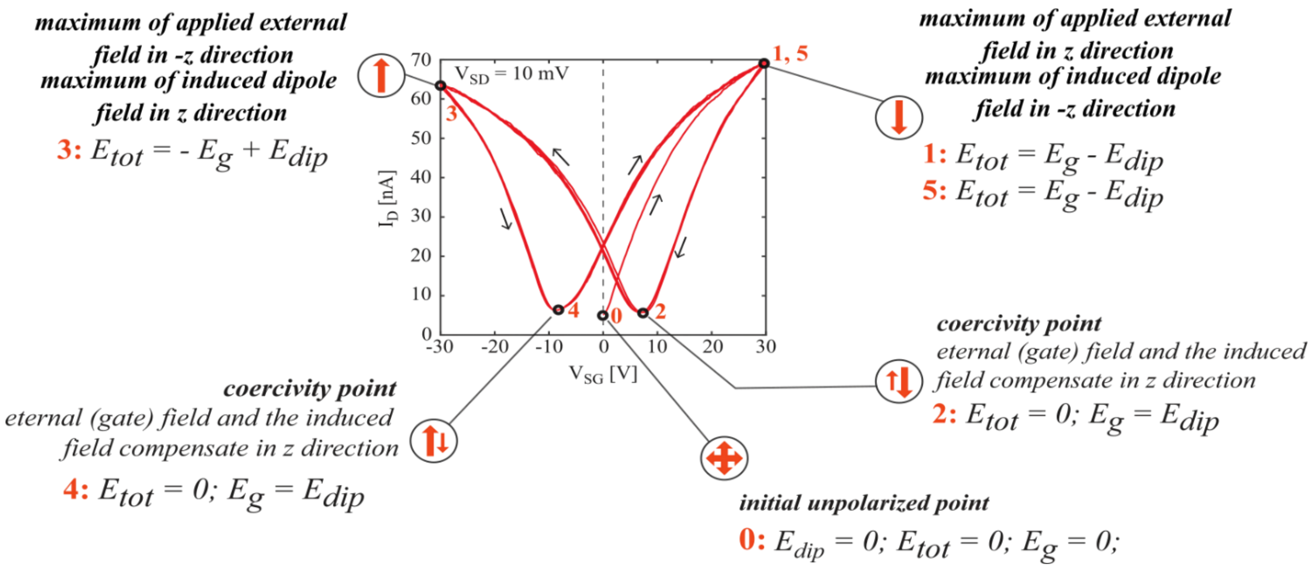

Figure S10: Elaboration of the hysteretic transfer curve presenting five subsequent sweeps at 300 K under low-vacuum, presenting 2L Gr NR FET. Prior to starting the measurements, the device was left under low-vacuum for over 24 h without biasing, so that the dipole states along the edges are depolarized. The points in the hysteresis loop labeled as 0-5, correspond to the following:

- "0": the initial unpolarized state
- "1": maximum of the -*z* polarized state (reaching maximum field in the positive *z* direction)
- "2": positive-side coercivity point, the position of the CNP indicates the strength of the induced dipole field
- "3": opposing polarity to "1"
- "4": negative-side coercivity point
- "5": repeated "1"

E_{tot} denotes the total mean field intensity in *z* direction experienced by the nanoribbon, and it is constituted of two components: applied external field by the global gate (E_g) and the induced water dipole field (E_{dip}). As the water dipoles are located on the sides of the ribbons, the resulting dipole field opposes E_g in *z* direction, yielding a reversed hysteresis loop.

S11: Device annealing sequence

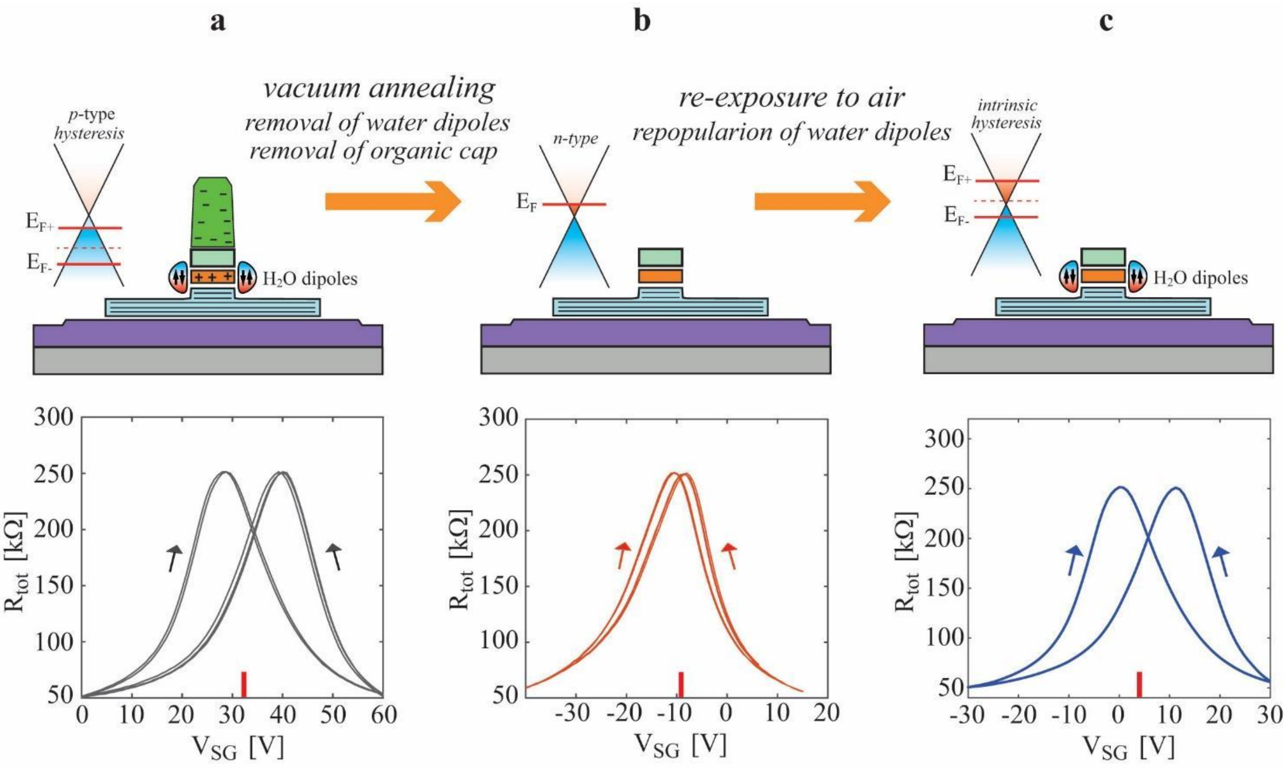

Figure S11: The total resistance transfer curves of a 1*L* Gr NR FET (measured under $2{\cdot}10^{-2}$ mbar, at 300 K). (a) the device prior to vacuum annealing at 520 K for 60 minutes under $8{\cdot}10^{-3}$ mbar, having pronounced *p*-type doping. (b) the transfer curve after vacuum annealing, and without breaking the vacuum of the chamber. (c) the same device measured under vacuum, after being exposed to the ambient conditions (relative humidity of 22-26 % at 298 K) for over 24 h. The red lines on the *x*-axis indicate the intrinsic (unpoled) position of the CNP. Schematic cross-sections above each transfer curve illustrate the sample structure and the likely source for the unintentional *p*-type doping in the device's initial state.

S12: Comparison of V_H in air and under vacuum

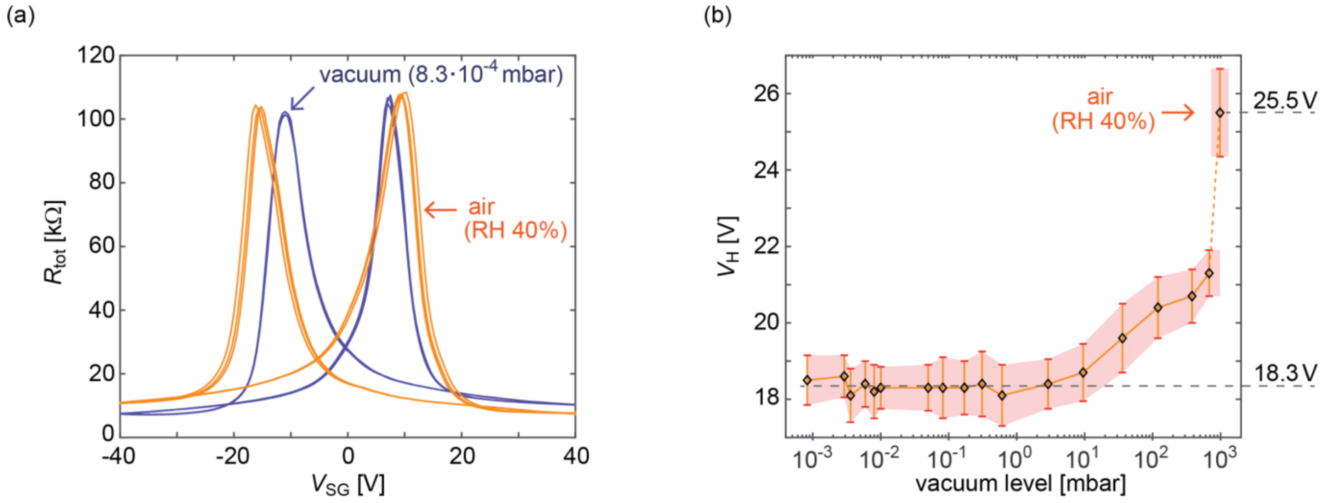

Figure S12: (a) electrical transfer curves (total device resistance; two-terminal) of a 1*L* Gr NR FET measured at 300 K in ambient pressure and 40 % relative humidity (RH), and immediately after under a vacuum of $8.3 \cdot 10^{-4}$ mbar. (b) for the same device, the dependence of the V_H on the vacuum conditions of the electrical measurement chamber. For each data point, V_H was extracted from the electrical transfer curves measured at 300 K with V_{SG} sweeping between -40 V and +40 V at a 2.5 Vs^{-1} rate. Vacuum level was controlled by adjusting the inlet of 40% RH air using a 0.1-200 sccm flow controller. The measurements were conducted in two ways: starting from the ambient and lowering the pressure, and starting from the lowest pressure and increasing to the ambient. Error bars indicate a standard deviation of the V_H from three subsequent sweeps. Going from 10^{-3} mbar to the ambient pressure and RH 40% the V_H opening increases by about 40 %, and the noticeable increase in V_H starts only after 10^1 mbar pressure.

S13: Mechanistic illustration of the self-stabilizing effect on the bi stable system

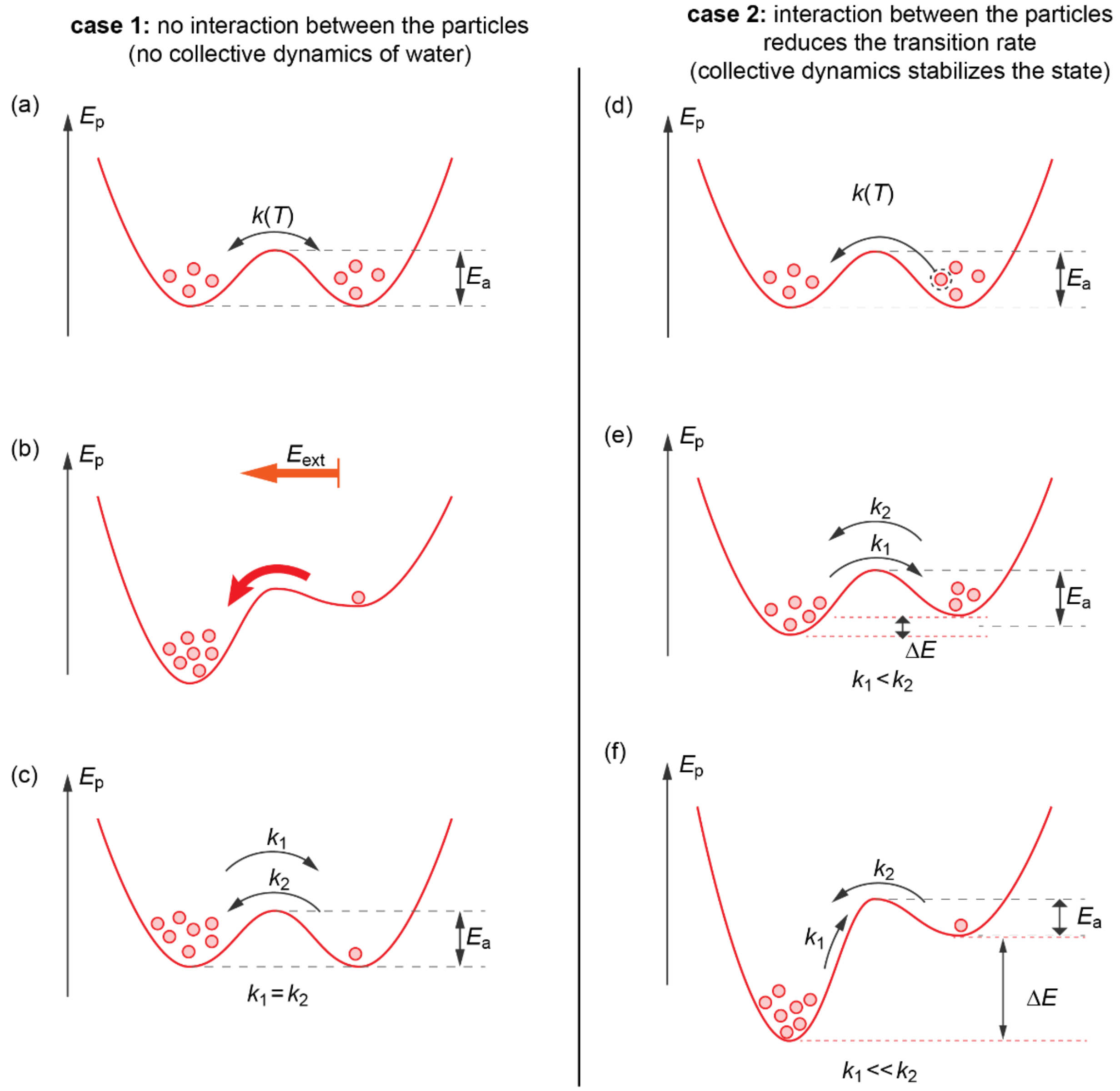

Figure S13. Mechanistic illustration of the self-stabilizing effect on the bi-stable system. (a) Potential energy profile (E_p) of a bi-stable system in equilibrium (equal distribution of the particles between the two states), with the exchange rate between the two states described by $k(T)$, and the energy barrier between the two states (E_a). (b) If the external field (E_{ext}) is applied, the potential profile is perturbed and one minimum is favored. (c) Upon canceling the E_{ext} the capability of the system to stay polarized depends on the ratio between the barrier height and the thermal energy of the particles. In (a-c) we do not consider interaction between the particles and even if one state is more occupied, the rate of crossing between the states is the same. In contrast, (d-f) considers that the interaction between the particles favors the state with higher occupation, *i.e.,* reduces the exchange rate. This would be the case when collective dynamics self-stabilizes the system in a polarized state. Let's assume that by thermal fluctuations one particle crosses over the barrier (d) and introduces non-equal occupation of the states. The interaction between the particles and the discrepancy in the occupation between the two states introduces the energy difference between the two minima ΔE and a more populated state is slightly more favored (e). Lastly, if we would polarize such an interacting system in an external field (as illustrated in (b)) and switch the external field off, being significantly more populated, one state would be significantly more favored (f). In this case, the exchange rates between the two states would significantly differ, and to de-polarize the system, a much larger effective barrier would be observed.

S14: Influence of the water molecule density on the induced field and water cluster stability

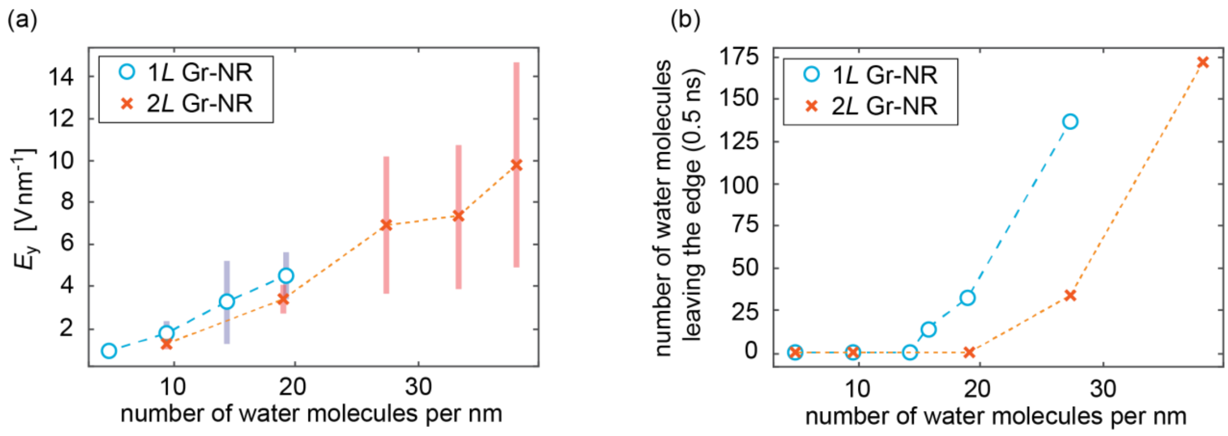

Figure S14. (a) Calculated strength of the dipole-induced field at the middle of 20 nm wide ribbons, as a function of the amount of water molecules present in the simulation and expressed per unit length of the edge. The model is relaxed in the external field of 2 V/nm in order to polarize the dipoles. (b) Number of water molecules that have detached from the edge after 0.5 ns of the simulation in a high external field of 4 V/nm, shown as a function of the number of water molecules per unit length of the edge. Circles and crosses represent the calculation results for 1*L* and 2*L* ribbons, respectively. For both sets of calculations, the temperature was set to 300 K. The error bars in (a) represent standard deviation from 20 independent relaxations of the model.

S15: Distribution of the water molecules along the ribbon edges

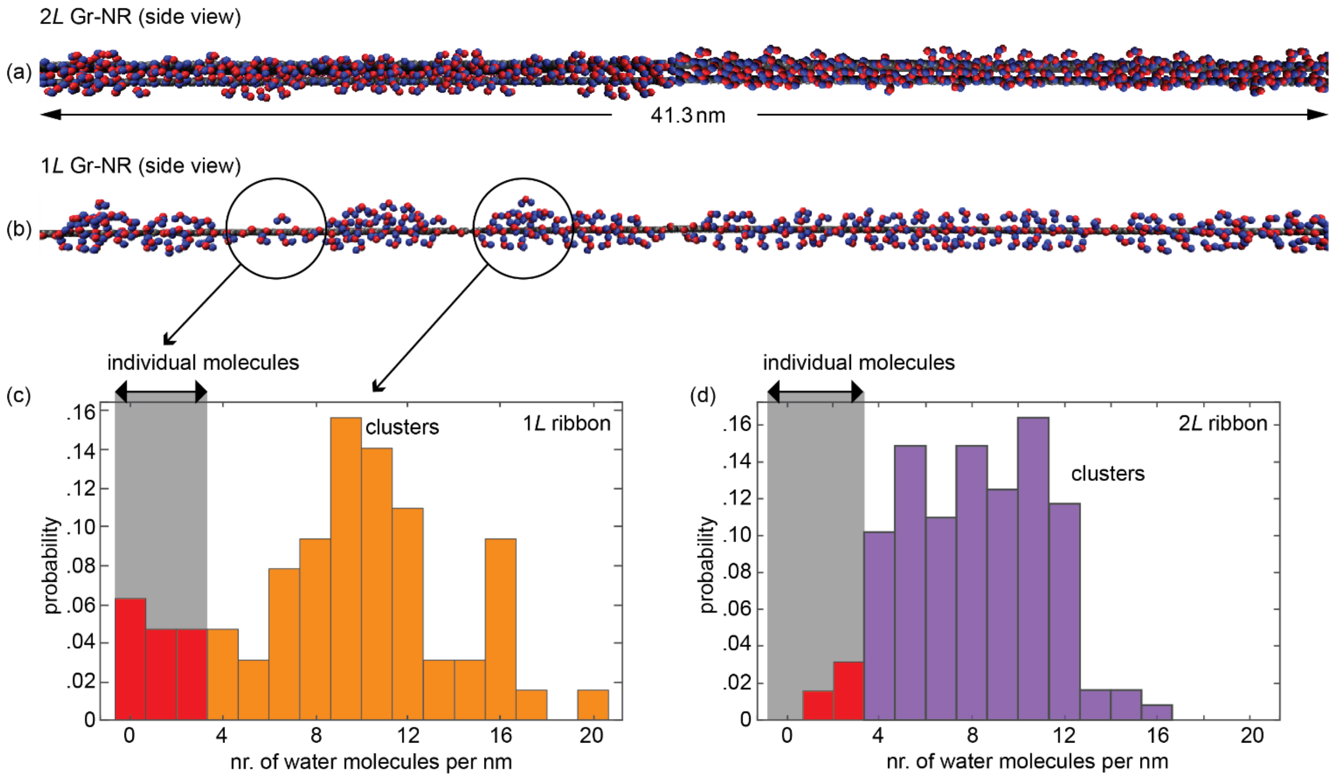

Figure S15. Side-view of the relaxed structure for (a) bi-layer ribbon, and (b) mono-layer ribbon. (c) and (d), probability of water cluster density for bi- and mono-layer ribbons, respectively. The data is extracted from a relaxed configuration at 300 K and without applying the external field. The results demonstrate two points: first, the formation of water clusters cannot be ruled out for the mono-layer ribbons, and second, individual water molecules adsorbed at the ribbon edges were essentially not found in the case of the bi-layers. Please, mind that the bars below 4 nm^{-1} correspond to the individually adsorbed molecules, and that for the bi-layers only the mobile water molecules are counted, not the "bridge" molecules between the layers.

S16: Estimate of the field enhancement due to the fringing capacitance effect

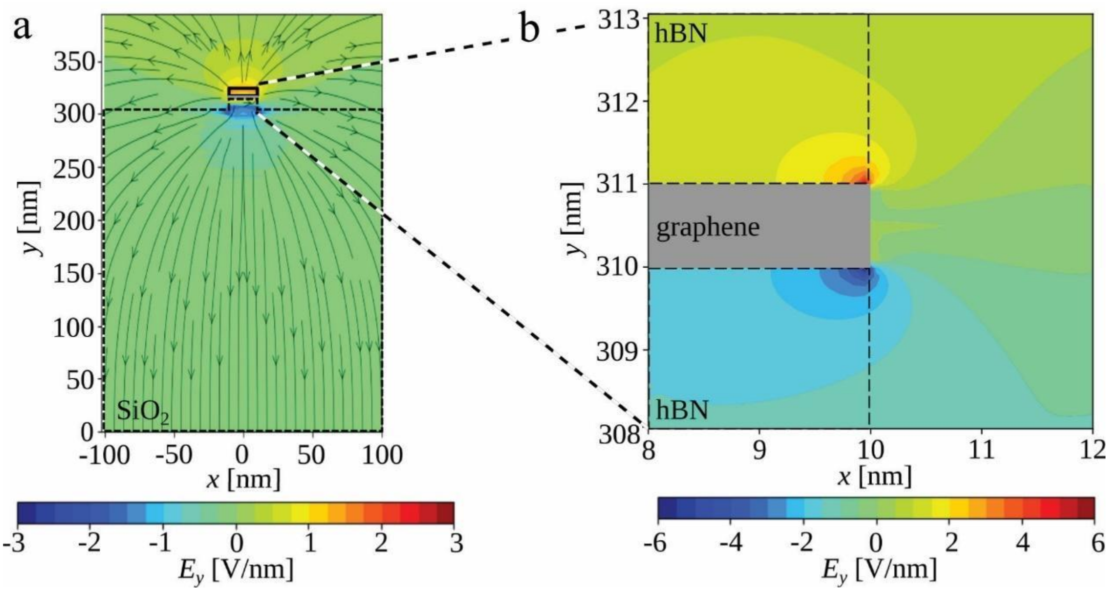

Figure S16: (a) an overview of the finite element model consisting of the conductive plane (at $y = 0$), SiO_2 layer and hBN/graphene/hBN nanoribbon. The model effectively describes a conductive wire above a conductive plane. 80 V bias is applied between the nanoribbon and the conductive plane. The field lines are depicted by black arrows, and the field intensity of the *y*-component is presented by colour-coding. (b) a zoom-in on one of the edges of the graphene nanoribbon, showing the y-component of the field and highlighting the field enhancement due to the fringing capacitance effect. At the edges, the fields up to 6 Vnm^{-1} are reached, and in the regions relevant to the water cluster the local fields are estimated to reach about 2 Vnm^{-1}. This yields ~8× field enhancement of the *y*-component of the externally applied field, in comparison to the parallel capacitance model.

S17: Field distribution from the MD simulation over the entire 2L NR width

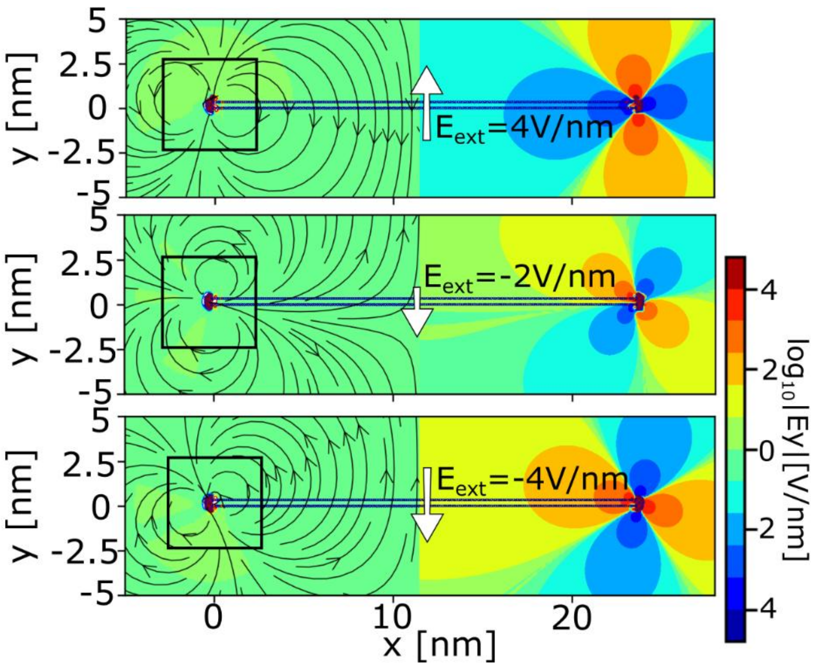

Figure S17: The field lines (left half) and the field strength (right half – semi-log scale), are showing only the induced fields by the dipoles. White arrow indicates the direction of the external field (field strength denoted), (top) shows positive saturation point, (middle) shows coercivity point, and (bottom) shows negative saturation point. The ribbon width is 25 nm. Zoom-in regions indicated by the solid black squares, and focusing to the water distribution along the edge are provided in Figure 3e-g of the main text.

S18. Overview of the MD model

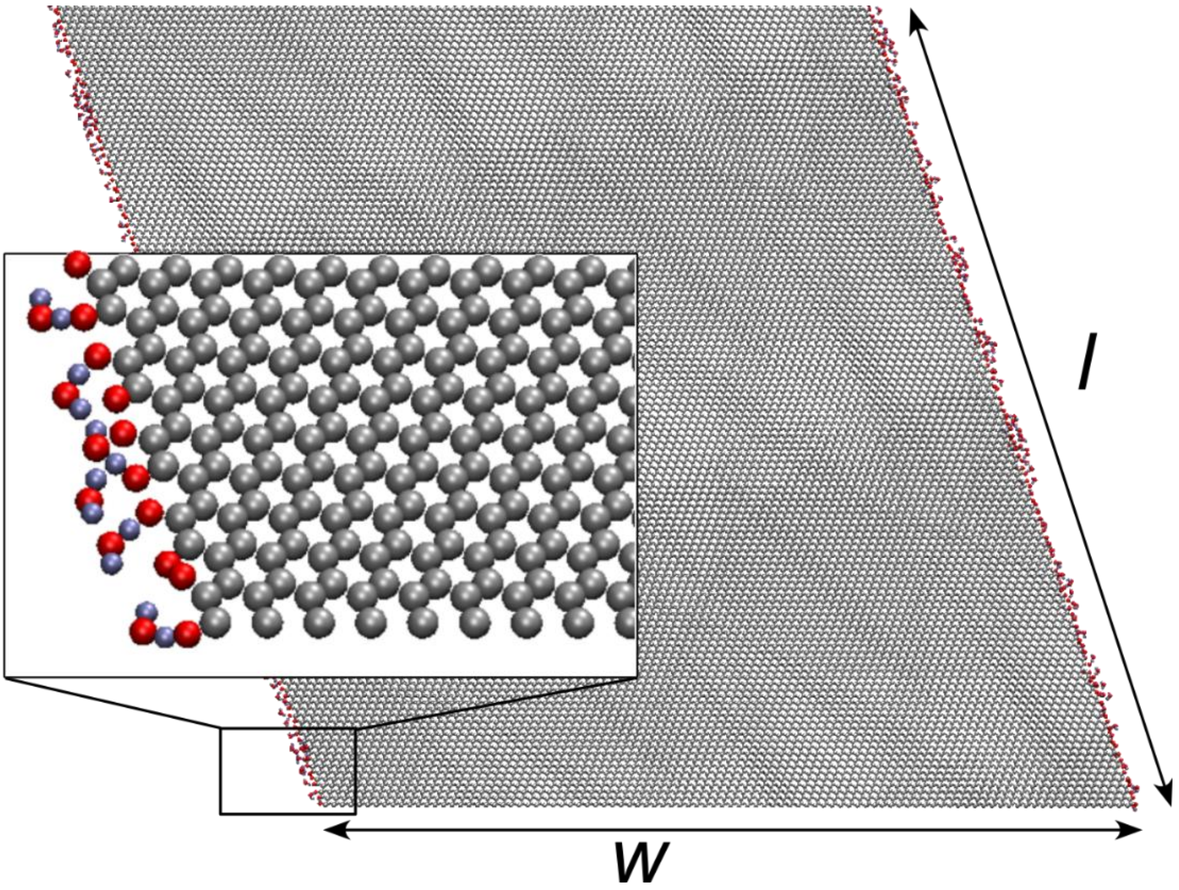

Figure S18: Full scale of the graphene nanoribbon model with water molecules on the edges. A total of 30720 carbon atoms were involved to yield the graphene mono-layer with 320 oxygen atoms on the edges. In the presented case the width (*W*) is 20 nm and length are 34 nm. A maximum of 2560 water molecules were simulated on the oxygen terminated edges.

S19: Dipole induced field decay as a function of the distance along the ribbon width direction

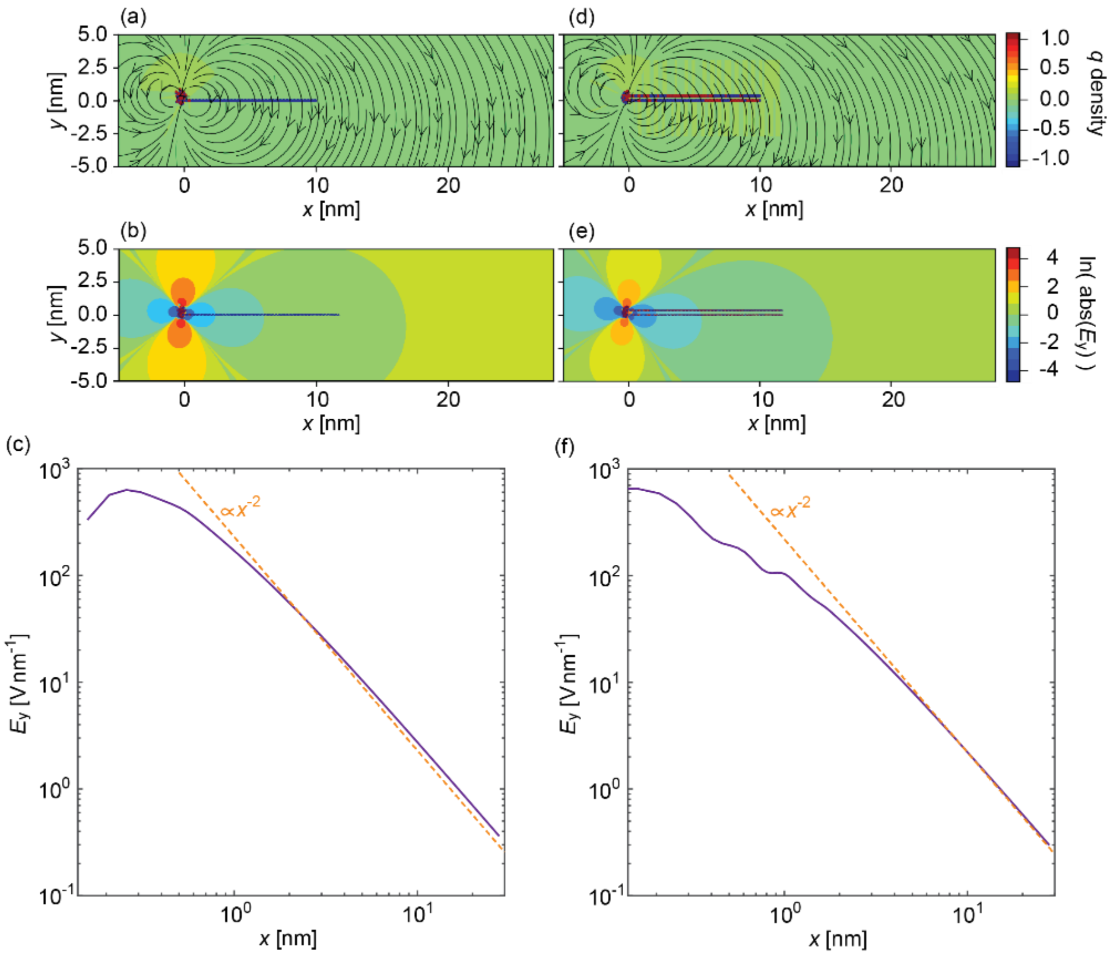

Figure S19: MD simulations of the field induced in *y*-direction (perpendicular to the basal plane) by the polarized dipoles (E_{ext} = 2 V/nm in the positive *y*-direction, 300 K) considering only one ribbon edge, in the case of 1*L* ribbon (a-c) and 2*L* ribbon (d-f). (a,d) Contour maps presenting the surface charge density. Electric field streamlines are superimposed to indicate the direction of electrostatic influence. The mirror-image placement of charges ensures lateral symmetry, and the color-coded regions emphasize strong confinement effects in the near-surface zone. (b,e) corresponding E_y field strength map (semi-log scale). (c,f) Intensity of the dipole-induced fields (solid purple lines) along the *x*-direction at 0.2 nm above the graphene plane, in log-log scale. A reference x^{-2} trend line (dashed orange) illustrates the asymptotic decay expected from a 1D line of dipoles, confirming consistency with analytical predictions.

S20. A comparison between symmetric and asymmetric gate bias sweeping.

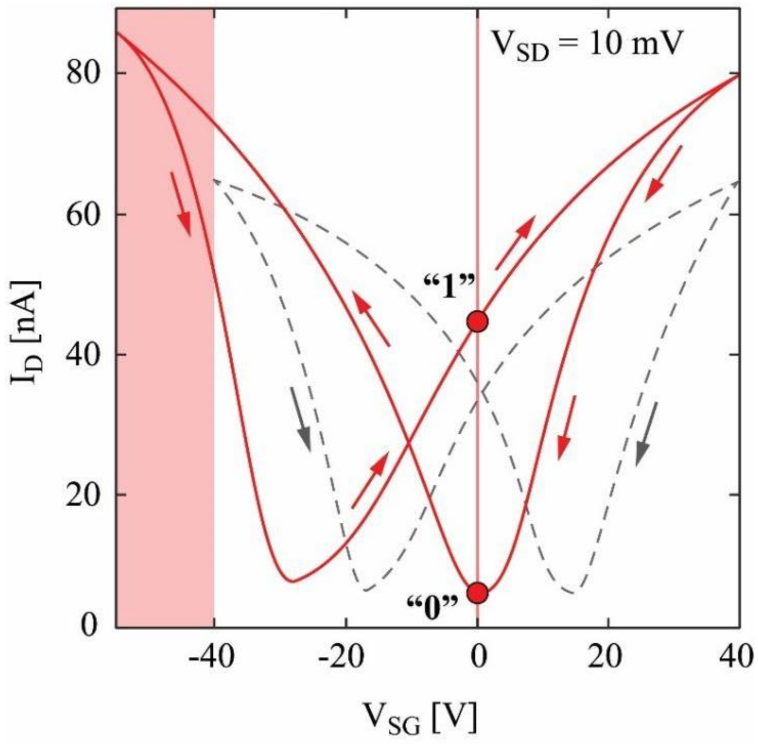

Figure S20: The same device (1*L* Gr NR FET, measured under $2\cdot10^{-2}$ mbar) is presented with two subsequent sweeping cycles repeated symmetrically and asymmetrically with respect to V_{SG} = 0 V. The asymmetric sweeps (solid red curves) are also presented in Figure 3a of the main text. Shaded red region indicates the extent of the asymmetric biasing needed to set CNP^{+} near V_{SG} = 0 V. In the case of the symmetric bias sweeping (dashed grey lines) the states "1" and "0" are degenerate in the I_D values, and therefore do not allow tracking of the field retention directly.
